# In vivo nucleus basalis of Meynert degeneration in mild cognitive impairment with Lewy bodies

**DOI:** 10.1016/j.nicl.2021.102604

**Published:** 2021-03-04

**Authors:** Julia Schumacher, John-Paul Taylor, Calum A. Hamilton, Michael Firbank, Ruth A. Cromarty, Paul C. Donaghy, Gemma Roberts, Louise Allan, Jim Lloyd, Rory Durcan, Nicola Barnett, John T. O'Brien, Alan J. Thomas

**Affiliations:** a Translational and Clinical Research Institute, Faculty of Medical Sciences, Newcastle University, Campus for Ageing and Vitality, Newcastle upon Tyne NE4 5PL, United Kingdom; b Nuclear Medicine Department, Newcastle upon Tyne Hospitals NFS Foundation Trust, Newcastle upon Tyne, United Kingdom; c Institute of Health Research, University of Exeter, Exeter, United Kingdom; d Department of Psychiatry, University of Cambridge School of Medicine, Cambridge CB2 0SP, United Kingdom

**Keywords:** Mild cognitive impairment, Alzheimer’s disease, dementia with Lewy bodies, Nucleus basalis of Meynert, EEG

## Abstract

**Objectives:**

To investigate *in vivo* degeneration of the cholinergic system in mild cognitive impairment with Lewy bodies (MCI-LB), we studied nucleus basalis of Meynert (NBM) volumes from structural MR images and its relation to EEG slowing and cognitive impairment.

**Methods:**

We studied the NBM using structural MR images in 37 patients with MCI-LB, 34 patients with MCI with Alzheimer’s disease (MCI-AD), and 31 healthy control participants. We also tested correlations between NBM volumes and measures of overall cognition and measures of EEG slowing in the MCI groups.

**Results:**

Overall NBM volume was reduced in MCI-LB compared to controls with no significant difference between MCI-AD and controls or between the two MCI groups. The voxel-wise analysis revealed bilateral clusters of reduced NBM volume in MCI-LB compared to controls and smaller clusters in MCI-AD compared to controls. There was a significant association between overall NBM volume and measures of overall cognition in MCI-LB, but not in MCI-AD. In both MCI groups, reduced NBM volume was correlated with more severe EEG slowing.

**Conclusions:**

This study provides *in vivo* evidence that early cholinergic degeneration in DLB occurs at the MCI stage and is related to the severity of cognitive impairment. Furthermore, the results suggest that early EEG slowing in MCI-LB might be in part cholinergically driven. Importantly, these findings suggest an early cholinergic deficit in MCI-LB that may motivate further testing of the effectiveness of cholinesterase inhibitors in this group.

## Highlights

• Nucleus basalis of Meynert (NBM) degeneration occurs early in Lewy body dementia.
• NBM degeneration is related to cognitive impairment in MCI with Lewy bodies.
• EEG slowing in MCI patients is related to the severity of NBM degeneration.

## 1 Introduction

Alzheimer’s disease (AD) and dementia with Lewy bodies (DLB) are characterized by marked cholinergic deficits (Lippa et al., 1999, Tiraboschi et al., 2002) which have been shown to be more severe in DLB compared to AD (Colloby et al., 2017, Hanyu et al., 2007, Kim et al., 2011, Whitwell et al., 2007) and are mainly caused by degeneration of the nucleus basalis of Meynert (NBM) (Erskine et al., 2019, Lippa et al., 1999, Perry et al., 1993). DLB is frequently preceded by a period of mild cognitive impairment (MCI) in which cognitive decline is present, but independence in activities of daily living is preserved (Donaghy et al., 2018, McKeith et al., 2020). In patients with Parkinson’s disease and AD, NBM degeneration occurs early and smaller NBM volumes are predictive of future cognitive impairment (Grothe et al., 2013, Grothe et al., 2012, Ray et al., 2018, Schulz et al., 2018, Teipel et al., 2018, Teipel et al., 2011). While post-mortem studies suggest that cholinergic deficits in DLB might occur earlier in the course of the disease compared to AD (Tiraboschi et al., 2002), no previous study has investigated early cholinergic changes in DLB *in vivo* and how these relate to cognitive impairment in patients with MCI with Lewy bodies (MCI-LB). The first aim of this study was therefore to investigate NBM volumes in MCI-LB compared to healthy controls and MCI with Alzheimer’s disease (MCI-AD) and the relationship with cognitive impairment in the MCI groups.

Quantitative EEG shows a slowing of the EEG rhythm in MCI-LB patients (Bonanni et al., 2015, van der Zande et al., 2020, Schumacher et al., 2020a). However, while early EEG abnormalities seem to be specific to Lewy body disease – they are much less severe in patients with MCI-AD – these changes are not observed in all MCI-LB patients (Schumacher et al., 2020a). The reason for this heterogeneity in EEG measures across patients is unclear and it is not clear what causes early EEG changes in MCI-LB. However, it has been suggested that EEG slowing in dementia patients might be related to cholinergic deficits (Adler et al., 2004, Babiloni et al., 2013, Riekkinen et al., 1991). Furthermore, we recently identified a relationship between a loss of EEG alpha reactivity and reduced NBM volumes in patients with Parkinson’s disease dementia (Schumacher et al., 2020b). The second aim of this study was therefore to investigate whether early changes in quantitative EEG measures – including measures of EEG slowing and alpha reactivity – are related to early NBM degeneration in MCI-LB.

## 2 Materials and methods

### 2.1 Participants

This study included 102 participants who were over 60 years of age. Recruitment and clinical assessment have been described previously (Schumacher et al., 2020a). Briefly, patients were recruited from local memory services and MCI diagnoses were made by a consensus panel of three experienced old-age psychiatrists according to NIA-AA criteria (Albert et al., 2011). In addition to a detailed clinical assessment, participants had already undergone dopaminergic imaging with ^123^I-N-fluoropropyl-2β-carbomethoxy-3β-(4-iodophenyl) single-photon emission computed tomography (FP-CIT SPECT) and ^123^iodine-metaiodobenzylguanidine (MIBG) myocardial scintigraphy through their involvement in an ongoing study investigating the diagnostic accuracy of imaging biomarkers in MCI and this information was used to apply diagnostic criteria (see below). All patients had a CDR of 0 or 0.5 and patients with a diagnosis of dementia or subjective cognitive impairment only were excluded.

The presence/absence of the core Lewy body symptoms (visual hallucinations, cognitive fluctuations, Parkinsonism, and REM sleep behaviour disorder) was rated by the panel utilizing the rating scales and all information from the clinical assessments (McKeith et al., 2017). A diagnosis of MCI with probable Alzheimer’s disease (MCI-AD) was given to patients who had no core Lewy body symptoms, negative FP-CIT and MIBG findings, and evidence of cognitive decline that was characteristic of AD, i.e. they met the additional NIA-AA criterion for “aetiology of MCI consistent with AD pathophysiologic process”. Probable MCI with Lewy bodies (MCI-LB) was diagnosed if a patient had two or more core Lewy body symptoms or one core symptom in addition to a positive FP-CIT and/or MIBG scan (McKeith et al., 2020).

According to these criteria, 37 participants were diagnosed with probable MCI-LB and 34 were diagnosed with MCI-AD. Eighteen MCI participants who were diagnosed with possible MCI-LB (i.e. only one core Lewy body symptom or a positive FP-CIT or MIBG scan) were excluded from the present analysis. Healthy controls (N = 31) were recruited from friends and relatives of the patients and from a local research register and had no history of psychiatric or neurological illness, no evidence of cognitive decline, and normal MR scans.

Written informed consent was obtained from all participants and the study was approved by the Newcastle & North Tyneside 2 Research Ethics Committee.

### 2.2 EEG acquisition and preprocessing

The resting state EEG protocol has been described before (Schumacher et al., 2020a). Briefly, 300 s of resting state eyes-closed EEG data followed by 300 s of resting state eyes-open EEG data were recorded using Waveguard caps (ANT Neuro) comprising 128 electrodes.

Preprocessing of eyes-closed and eyes-open EEG data was performed using the EEGLAB toolbox (version 14) in Matlab (R2017a) and included bandpass-filtering between 0.3 and 54 Hz, creating non-overlapping epochs of 2 s, visually inspecting the data, and exclusion of noisy channels and epochs (mean (standard deviation) number of excluded channels, HC: 6.4 (2.8), MCI-AD: 8.3 (4.1), MCI-LB: 6.5 (3.2)) (Schumacher et al., 2020a). Subsequently, independent component analysis was applied and components representing muscular, cardiac, ocular, or electrical (50 Hz line noise) artefacts were rejected. Previously excluded channels were replaced using spherical spline interpolation and data were average referenced. For each participant, the first 45 2-second long artefact-free epochs of eyes-closed and the first 45 2-second long epochs of eyes-open EEG data were selected for analysis.

### 2.3 Quantitative analysis of eyes-closed EEG data

The quantitative EEG analysis for eyes-closed data has been detailed previously (Schumacher et al., 2020a). Briefly, the power spectral density (PSD) was estimated for each 2-second epoch and mean power across all included epochs was estimated for different standard EEG frequency bands including delta (2–4 Hz), theta (4–5.5 Hz), pre-alpha (5.5–8 Hz), alpha (8–13 Hz), and beta (13–30 Hz). The pre-alpha band was included based on previous studies in DLB (Bonanni et al., 2008). The PSD within each frequency band was normalized by the total power across the power spectrum. The dominant frequency (DF) was calculated as the frequency with the highest power between 4 and 15 Hz (averaged across epochs). Dominant frequency was calculated for all electrodes as well as from occipital electrodes only (PO9, PO7, POO9h, PO5, O1, PO3, POO3h, OI1h, POz, Oz, PO4, POO4h, PO6, O2, OI2h, PO8, POO10h, PO10). Results from this analysis have been reported previously (Schumacher et al., 2020a).

### 2.4 EEG alpha reactivity analysis

EEG data from three occipital electrodes (O1, O2, and Oz) were used for the alpha reactivity analysis (Schumacher et al., 2020b, Wan et al., 2019). The PSD for the three electrodes was averaged across all included epochs and across the three electrodes for each condition separately (eyes-open and eyes-closed).

Alpha reactivity was calculated according to the following formula (Wan et al., 2019):

$$alpha\;reactivity=\frac{alpha\;power\;eyes\;closed-alpha\;power\;eyes\;open}{alpha\;power\;eyes\;closed}$$

where alpha power was computed as the relative power within a frequency bin around the individual alpha peak frequency ±2 Hz. Individual alpha peak frequencies were calculated by locating the peak in the PSD in the extended alpha frequency band from 4 to 14 Hz (Babiloni et al., 2017, Babiloni et al., 2011) using the eyes-closed data. Individual alpha peak frequencies were used instead of the standard alpha frequency band to account for a shift of the alpha peak to slower frequencies in MCI patients (Bonanni et al., 2015, Schumacher et al., 2020a).

### 2.5 MRI acquisition and preprocessing

MR images were acquired on a 3T Philips Intera Achieva scanner with a magnetisation prepared rapid gradient echo (MPRAGE) sequence, sagittal acquisition, echo time 4.6 ms, repetition time 8.3 ms, inversion time 1250 ms, flip angle = 8°, SENSE factor = 2, and in-plane field of view 216 × 240 mm^2^ with slice thickness 1.0 mm, yielding a voxel size of 1.0 × 1.0 × 1.0 mm^3^.

Preprocessing of MR images was performed in SPM12 (http://www.fil.ion.ucl.ac.uk/spm/). Images were first segmented into grey matter, white matter, and cerebrospinal fluid. The DARTEL algorithm (Ashburner, 2007) was used to create a study-specific template and all grey matter images were coregistered to this template. As a final step, images were smoothed with a 4 mm full width at half maximum Gaussian kernel.

### 2.6 NBM volume analysis

**Fig. 1:**
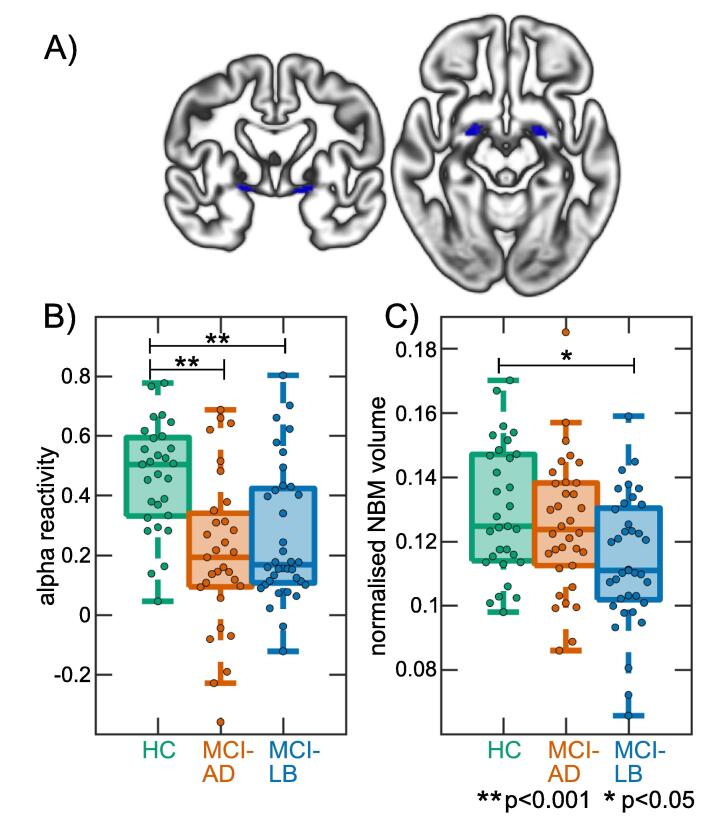
NBM region of interest and group comparisons. A) NBM mask overlaid on the study-specific DARTEL grey matter template. B) Comparison of alpha reactivity between groups. C) Comparison of overall NBM volume between groups. HC, healthy controls; MCI-AD, mild cognitive impairment with Alzheimer’s disease; MCI-LB, mild cognitive impairment with Lewy bodies; NBM, nucleus basalis of Meynert.

The NBM forms part of the basal forebrain which consists of cholinergic cells that can be histologically defined as Ch1-Ch6 where Ch4 corresponds to the NBM (Mesulam et al., 1983). A region of interest mask of the NBM (see Fig. 1A) was created using a probabilistic anatomical map from the SPM Anatomy Toolbox (Eickhoff et al., 2005) which is based on microscopic delineations of ten post-mortem human brains (Zaborszky et al., 2008). The mask consisted of 71 voxels for the left NBM and 59 voxels for the right NBM. To transform the NBM mask from MNI standard space to the study-specific template space, we used Advanced Normalization Tools (Avants et al., 2011, Klein et al., 2009) to affine register the study-specific DARTEL template to MNI space and applied the inverse transform to the NBM map.

For each participant, using the unsmoothed grey matter images, grey matter volume within the NBM mask was calculated and averaged across right and left hemispheres. Total intracranial volume was calculated in SPM and was used to normalise NBM volumes using proportional normalisation.

### 2.7 Statistics

EEG alpha reactivity and overall NBM volume were compared between the groups using univariate ANOVAs including covariates for age and sex. This was followed by post-hoc tests which were Bonferroni-corrected for multiple comparisons.

In addition to investigating overall NBM volumes, we also conducted a voxel-wise group comparison of volumes within the NBM mask by running a voxel-based morphometry (VBM) analysis in SPM using the smoothed grey matter images. We included covariates for age, sex, and total intracranial volume. The statistical threshold was set at p < 0.05, family wise error (FWE)-corrected for multiple comparisons.

In the MCI-AD and MCI-LB groups separately, Spearman’s correlations were calculated between the different EEG characteristics and overall NBM volume. Additionally, the association between NBM volume and the Mini Mental State Examination (MMSE) and Addenbrooke’s Cognitive Examination - Revised (ACE-R) as measures of global cognitive impairment were assessed using Spearman’s correlations in the two MCI groups separately. All p-values from the correlation analyses were false discovery rate (FDR)-corrected for multiple comparisons.

Furthermore, the MCI-LB group was dichotomized with respect to the presence (N = 17) vs absence (N = 19) of treatment with cholinesterase inhibitors, and NBM volumes were compared between the subgroups using two-sample *t*-tests.

## 3 Results

### 3.1 Demographics

All three groups were similar in age and the two MCI groups were similar in terms of global cognitive impairment (Table 1). There were more male participants in the MCI-LB group whereas the MCI-AD group involved more female participants. As expected, the MCI-LB group had more Parkinsonism, and higher cognitive fluctuation and visual hallucination scores compared to the MCI-AD group and more MCI-LB participants were taking cholinesterase inhibitors and Parkinson’s medication.

**Table 1:** Demographic and clinical variables, mean (standard deviation).

	HC (N = 31)	MCI-AD (N = 34)	MCI-LB (N = 37)	Group differences
Male: female	22:9	15:19	33:4	χ^2^ = 16.8, p < 0.001^a^ p(HC,MCI-AD) = 0.03 p(HC,MCI-LB) = 0.06 p(MCI-AD,MCI-LB) < 0.001
Age	73.7 (7.3)	76.3 (7.6)	74.7 (6.5)	F(2,99) = 1.1, p = 0.33^b^
AChEI	–	6 (18%)^e^	17 (46%)^f^	χ^2^ = 6.1, p = 0.01^c^
PD meds	–	0^e^	4 (11%)^f^	χ^2^ = 3.8, p = 0.052^c^
Years of education	14.7 (4.0)^g^	12.7 (3.3)^h^	12.0 (2.9)	F(2,96) = 5.5, p = 0.005^c^ p(HC,MCI-AD) = 0.06 p(HC,MCI-LB) = 0.005 p(MCI-AD,MCI-LB) = 1.0
ACE-R	92.7 (4.2)	82.3 (8.7)	83.5 (9.3)	t_69_ = 0.6, p = 0.58^d^
MMSE	28.5 (1.1)	26.9 (2.2)	26.5 (2.5)	t_69_ = 0.9, p = 0.39^d^
UPDRS III	5.5 (4.4)	15.7 (14.0)	23.5 (14.5)	t_69_ = 2.3, p = 0.02^d^
DCFS	–	6.9 (1.9)^i^	8.4 (3.3)^k^	t_59_ = 2.2, p = 0.03^d^
CAF total	–	1.4 (2.7)^i^	3.8 (4.3)^k^	t_59_ = 2.5, p = 0.01^d^
NPI total	–	8.6 (9.3)^i^	16.1 (13.1)^k^	t_59_ = 2.5, p = 0.02^d^
NEVHI	–	0.8 (1.6)^e^	2.9 (4.2)	t_67_ = 2.6, p = 0.01^d^

ACE-R, Addenbrooke’s Cognitive Examination – Revised; AChEI, number of patients taking acetylcholinesterase inhibitors; CAF total, Clinician Assessment of Fluctuation total score; DCFS, Dementia Cognitive Fluctuation Scale; HC, healthy controls; MCI-AD, mild cognitive impairment with Alzheimer’s disease; MCI-LB, mild cognitive impairment with Lewy bodies; MMSE, Mini Mental State Examination; NEVHI, North-East Visual Hallucinations Interview; NPI, Neuropsychiatric Inventory; PD meds, number of patients taking dopaminergic medication for the management of Parkinson’s disease symptoms; UPDRS III, Unified Parkinson’s Disease Rating Scale III (motor subsection)

a Chi-square test HC, MCI-AD, MCI-LB; ^b^ Univariate ANOVA HC, MCI-AD, MCI-LB; ^c^ Chi-square test MCI-AD, MCI-LB; ^d^ Student’s *t*-test MCI-AD, MCI-LB.

e N = 32, ^f^ N = 36, ^g^ N = 29, ^h^ N = 33, ^i^ N = 27, ^k^ N = 34

### 3.2 EEG frequency analysis

The results of the frequency analysis have been reported previously (Schumacher et al., 2020a). In summary, there was a general slowing of the EEG in MCI-LB patients compared to healthy controls and MCI-AD by a shift in power from beta and alpha frequency bands towards slower frequencies in the pre-alpha and theta range. This was also reflected by a shift of the dominant frequency towards slower frequencies in the MCI-LB group compared to MCI-AD and controls.

### 3.3 EEG alpha reactivity analysis

Two control participants, three MCI-AD, and one MCI-LB patient had to be excluded from the alpha reactivity analysis because their eyes-open EEG data were too noisy. The alpha reactivity analysis therefore included 29 controls, 31 MCI-AD, and 36 MCI-LB participants.

There was an overall effect of diagnosis on alpha reactivity (F(2,91) = 11.2, p < 0.001, Fig. 1B). Post-hoc tests showed that alpha reactivity was significantly reduced in MCI-AD compared to controls (p < 0.001) and in MCI-LB compared to controls (p = 0.008) whereas there was no significant difference between the two MCI groups (p = 0.30).

### 3.4 NBM volume analysis

The ANOVA showed an effect of diagnosis on overall NBM volume (F(2,97) = 4.2, p = 0.02). Post-hoc tests revealed that overall NBM volumes were significantly reduced in the MCI-LB group compared to controls (p = 0.01) with no significant difference between MCI-AD and controls (p = 0.49) or between the two MCI groups (p = 0.53, Fig. 1C).

Including a covariate for cholinesterase inhibitor use did not change the results: F(2,92) = 4.4, p = 0.02; p(HC,MCI-AD) = 0.66, p(HC,MCI-LB) = 0.01, p(MCI-AD,MCI-LB) = 0.26.

**Fig. 2:**
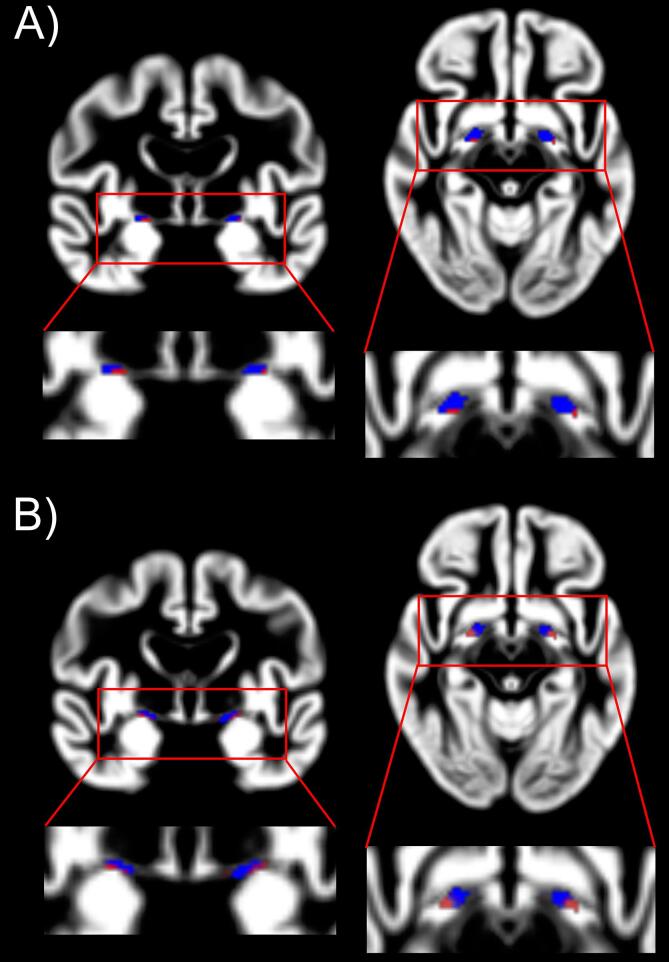
Voxel-based comparison of NBM volumes. A) Significant clusters within the NBM mask for the contrast HC > MCI-AD. B) Significant clusters within the NBM mask for the contrast HC > MCI-LB. The NBM mask is shown in blue and significant voxels are shown in red. See Table 2 for statistics of significant clusters. (For interpretation of the references to colour in this figure legend, the reader is referred to the web version of this article.) HC, healthy controls; MCI-AD, mild cognitive impairment with Alzheimer’s disease; MCI-LB, mild cognitive impairment with Lewy bodies; NBM, nucleus basalis of Meynert

The voxel-wise analysis revealed a bilateral reduction in NBM volume in the MCI-LB group compared to controls (Table 2, Fig. 2A), with about 27% of the right and 17% of the left NBM showing more severe atrophy in the MCI-LB group compared to controls. Smaller clusters of reduced NBM volume in the MCI-AD group compared to controls were found bilaterally (Table 2, Fig. 2B), with about 8% of the left NBM and about 7% of the right NBM showing more severe atrophy in MCI-AD compared to controls. There were no significant clusters with increased NBM volume in either MCI group compared to controls and no significant differences in the direct comparison between MCI-AD and MCI-LB. The results from the comparison of the two MCI groups did not change when including a covariate for the use of cholinesterase inhibitors.

**Table 2:** Significant clusters (p_FWE_ < 0.05) from voxel-based morphometry analysis. Cluster size is given in voxels. There were no significant clusters for the other contrasts (MCI-AD > HC and MCI-LB > HC) or for the comparison between the two MCI groups.

	cluster size	T	p (FWE)	location
*HC > MCI-AD*				
	4	3.51	0.042	right NBM
	5	3.39	0.039	left NBM

*HC > MCI-LB*				
	13	3.79	0.032	right NBM
	9	3.77	0.037	left NBM
	3	3.34	0.037	right NBM
	3	3.32	0.042	left NBM

### 3.5 Association between EEG measures and NBM volume

**Fig. 3:**
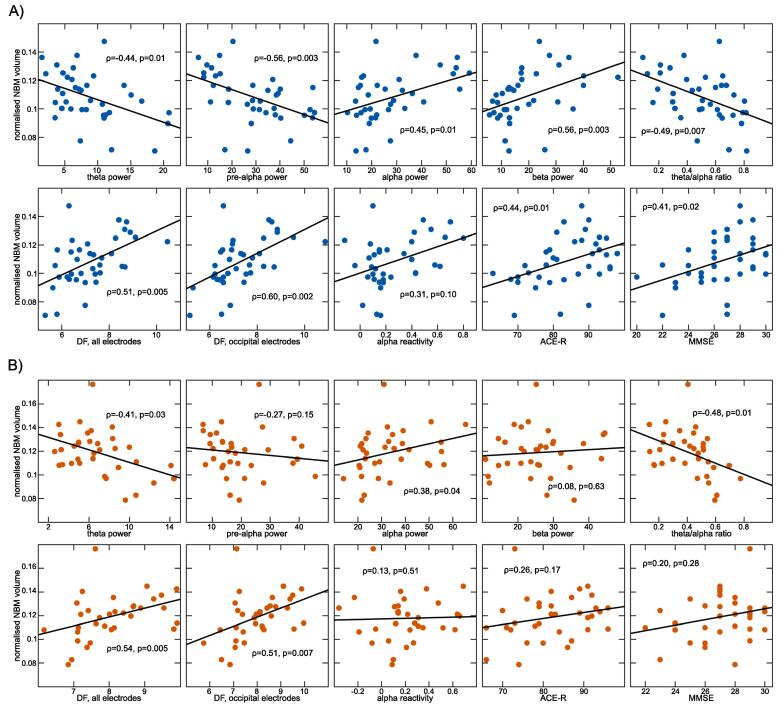
Relationship between NBM volume and EEG slowing and cognitive impairment. Correlations between NBM volume and EEG and cognitive measures in A) MCI-LB and B) MCI-AD. All p-values are FDR-corrected for multiple comparisons. ACE-R, Addenbrooke’s Cognitive Examination – Revised; DF, dominant frequency; MCI-AD, mild cognitive impairment with Alzheimer’s disease; MCI-LB, mild cognitive impairment with Lewy bodies; MMSE, Mini-Mental State Examination; NBM, nucleus basalis of Meynert.

In the MCI-LB group, theta and pre-alpha power and the theta/alpha ratio were negatively and alpha and beta power were positively correlated with overall NBM volume (Table 3 and Fig. 3A). Furthermore, dominant frequency was positively correlated with NBM volume. In the MCI-AD group, theta power and the theta/alpha ratio were negatively correlated with NBM volume whereas alpha power and dominant frequency were positively correlated with NBM volume (Table 3 and Fig. 3B). There was no significant correlation between NBM volume and alpha reactivity in either group.

**Table 3:** Spearman’s correlations between different quantitative EEG characteristics, cognitive variables, and mean NBM volume in MCI-AD and MCI-LB. P-values are FDR-corrected for multiple comparisons.

	MCI-AD	MCI-LB
Delta power	ρ = −0.27, p = 0.15	ρ = −0.29, p = 0.12
Theta power	ρ = −0.41, p = 0.03	ρ = −0.44, p = 0.01
Pre-alpha power	ρ = −0.27, p = 0.15	ρ = −0.56, p = 0.003
Alpha power	ρ = 0.38, p = 0.04	ρ = 0.45, p = 0.01
Beta power	ρ = 0.08, p = 0.63	ρ = 0.56, p = 0.003
Theta/alpha ratio	ρ = −0.48, p = 0.01	ρ = −0.49, p = 0.007
DF, all electrodes	ρ = 0.54, p = 0.005	ρ = 0.51, p = 0.005
DF, occipital electrodes	ρ = 0.51, p = 0.007	ρ = 0.60, p = 0.002
Alpha reactivity	ρ = 0.13, p = 0.51	ρ = 0.31, p = 0.10
ACE-R	ρ = 0.26, p = 0.17	ρ = 0.44, p = 0.01
MMSE	ρ = 0.20, p = 0.28	ρ = 0.41, p = 0.02

ACE-R, Addenbrooke’s Cognitive Examination – Revised; DF, dominant frequency; MCI-AD, mild cognitive impairment with Alzheimer’s disease; MCI-LB, mild cognitive impairment with Lewy bodies; MMSE, Mini-Mental State Examination

### 3.6 Clinical correlations

In MCI-LB, NBM volume was positively correlated with the MMSE score and with the ACE-R whereas there were no significant correlations in the MCI-AD group between NBM volume and MMSE or ACE-R scores (Table 3 and Fig. 3).

There were no differences between MCI-LB patients who were taking cholinesterase inhibitors compared to those who were not in terms of overall NBM volume (t_34_ = 0.57, p = 0.57).

## 4 Discussion

In this study, we showed that atrophy of the NBM in MCI-LB is greater than normal age-related volume loss and occurs bilaterally with about 20% of the NBM showing significantly more severe atrophy compared to controls. Previous studies have reported degeneration of the NBM in DLB patients at the dementia stage (Colloby et al., 2017, Hanyu et al., 2007, Kim et al., 2011, Whitwell et al., 2007) and findings from a post-mortem study have suggested that cholinergic deficits might occur early in the course of the disease (Tiraboschi et al., 2002). The results of the present study support this finding by providing *in vivo* evidence of early cholinergic system alterations in DLB.

In MCI-AD, while overall NBM volumes were not significantly different from healthy controls, the voxel-wise analysis revealed atrophy in similar locations as in the MCI-LB group, albeit with smaller spatial extent. This is in line with previous studies that have reported degeneration of the NBM in MCI-AD compared to healthy controls (Grothe et al., 2013, Grothe et al., 2012, Teipel et al., 2011).

While the direct comparison between the MCI groups did not reveal significant differences, the voxel-wise comparison with healthy control volumes suggests that the spatial extent of NBM atrophy in MCI-LB might be greater than in MCI-AD. This would be in line with previous reports of more severe structural abnormalities within the cholinergic system in DLB compared to AD (Colloby et al., 2017, Hanyu et al., 2005, Kim et al., 2011, Tiraboschi et al., 2002) and provides tentative indication that this difference might already be evident in early stages of the disease.

Reduced NBM volumes were correlated with more severe EEG slowing in both MCI groups, suggesting that the cholinergic system might have an influence on the resting state cortical EEG signal in MCI patients. In the MCI-LB group, a greater shift of power towards slower frequencies and a more severe slowing of the dominant EEG rhythm were related to more severe NBM degeneration whereas in the MCI-AD group, this relationship was mainly restricted to the dominant EEG rhythm. These results are in line with previous studies at the dementia stage suggesting that EEG slowing in dementia patients might be related to cholinergic degeneration (Adler et al., 2004, Babiloni et al., 2013, Riekkinen et al., 1991). Our study suggests that this relationship is already evident in patients at the MCI stage. While early EEG slowing shows relatively high specificity for MCI-LB over MCI-AD, a significant number of MCI-LB patients still exhibit EEG patterns which are similar to healthy control and MCI-AD levels (Schumacher et al., 2020a). The present results indicate that the level of cholinergic degeneration might play a role in explaining this heterogeneity, suggesting that MCI-LB patients with less early NBM atrophy might show fewer abnormalities in quantitative EEG measures.

EEG alpha reactivity which has been suggested as a marker of cholinergic system integrity (Osipova et al., 2003, Wan et al., 2019) was not related to NBM volumes in either MCI group. This is in line with our previous investigation of alpha reactivity in dementia patients where we found an association with NBM volumes only in patients with Parkinson’s disease dementia, but not in AD or DLB (Schumacher et al., 2020b). Patients with an initial presentation of Parkinsonism were excluded from the present study. Our results therefore further support the finding that NBM degeneration does not seem to be related to a loss of alpha reactivity in AD or DLB patients.

Smaller NBM volumes were related to more severe cognitive impairment in MCI-LB whereas there was no significant association between cognitive impairment and NBM volumes in the MCI-AD group. This suggests that the early cholinergic deficit observed in MCI-LB might be more directly and more strongly related to cognitive impairment whereas in MCI-AD there might be additional factors that are driving cognitive impairment at this disease stage, such as neurodegeneration of the medial temporal lobe (Kantarci et al., 2016, Mak et al., 2016). In turn, this also suggests that cholinergic remediation might provide a larger improvement in cognitive function in MCI-LB patients, and that EEG may help to stratify those patients who might respond to treatment at this early stage.

A potential limitation of the present study is the use of cholinesterase inhibitors in the MCI patients and more MCI-LB patients were taking these medications compared to the MCI-AD group which might have influenced group comparisons of NBM volumes or EEG measures (Gianotti et al., 2008). Such prescribing reflects local use for neuropsychiatric symptoms in Lewy body diseases and is in line with recent advice (Taylor et al., 2020). However, we did not find differences in NBM volumes between MCI-LB patients who were taking cholinesterase inhibitors compared to those patients not taking these medications and including cholinesterase inhibitor use as a covariate in the group comparisons did not change the results. Similarly, when previously comparing MCI patients who were taking cholinesterase inhibitors to those who were not, we did not find any significant differences in any EEG measures (Schumacher et al., 2020a). However, investigating the prospective effect of cholinesterase inhibitor treatment on NBM volumes in MCI-LB patients in more detail will be an interesting avenue for future research (Cavedo et al., 2017). Four patients from the MCI-LB group were also taking dopaminergic medication for the management of Parkinson’s disease symptoms (three patients were taking Carbidopa-Levodopa and one patient was taking Rotigotine). However, the small number of patients taking these medications did not allow for a more in-depth analysis of the effect of dopaminergic medication on cognition, NBM volumes or EEG measures (Babiloni et al., 2019); this remains a limitation of this work.

Another possible limitation is the fact that the majority of our MCI-LB patients were male while the male/female ratio was more balanced in the MCI-AD group. This might have influenced our results given that there is some evidence for sex differences in NBM structure (Amunts, 2007). Sex imbalances are an inherent problem of many AD/DLB comparison studies due to the higher prevalence of DLB in men and AD in women, respectively (Beam et al., 2018, Savica et al., 2013). To minimise the impact of these group imbalances on our results, we have included a covariate for sex in all analyses. Nevertheless, studying the influence of sex on NBM degeneration in MCI patients will be an important step in future studies.

In conclusion, this study provides *in vivo* insight into early pathophysiology in Lewy body disease by suggesting that early degeneration of the cholinergic system occurs in MCI-LB patients and is related to the severity of cognitive impairment. Furthermore, the results suggest that early EEG slowing in MCI patients might be cholinergically driven and that differences in the level of cholinergic deficits might explain heterogeneity with respect to EEG findings in MCI-LB patients. Importantly, these findings suggest that the cholinergic system is affected early in MCI-LB and thus underline the potential effectiveness of early treatment with cholinesterase inhibitors in these patients.

## Funding

This research was funded by 10.13039/501100002283Alzheimer’s Research UK (ARUK-PG2015-13) and supported by the 10.13039/501100012295NIHR Newcastle Biomedical Research Centre (grant numbers BH120812 and BH120878). GE Healthcare provided the FP-CIT radioligand for this investigator-led study.

## CRediT authorship contribution statement

**Julia Schumacher:** Conceptualization, Methodology, Software, Formal analysis, Visualization, Writing - original draft. **John-Paul Taylor:** Conceptualization, Supervision, Project administration, Funding acquisition, Writing - review & editing. **Calum A. Hamilton:** Investigation, Writing - review & editing. **Michael Firbank:** Investigation, Data curation, Project administration, Funding acquisition, Writing - review & editing. **Ruth A. Cromarty:** Investigation, Writing - review & editing. **Paul C. Donaghy:** Investigation, Writing - review & editing. **Gemma Roberts:** Investigation, Writing - review & editing. **Louise Allan:** Conceptualization, Project administration, Funding acquisition, Writing - review & editing. **Jim Lloyd:** Conceptualization, Project administration, Funding acquisition, Writing - review & editing. **Rory Durcan:** Investigation, Writing - review & editing. **Nicola Barnett:** Investigation, Writing - review & editing. **John T. O’Brien:** Conceptualization, Project administration, Funding acquisition, Writing - review & editing, Conceptualization, Project administration, Funding acquisition, Writing - review & editing. **Alan J. Thomas:** Conceptualization, Project administration, Funding acquisition, Writing - review & editing.

## Declaration of Competing Interest

The authors declare that they have no known competing financial interests or personal relationships that could have appeared to influence the work reported in this paper.
